# Applicability of the Theory of Planned Behavior for Predicting Alcohol Use in Spanish Early Adolescents

**DOI:** 10.3390/ijerph17228539

**Published:** 2020-11-18

**Authors:** Olalla Cutrín, Isotta Mac Fadden, Stephanie L. Ayers, Stephen S. Kulis, Jose Antonio Gómez-Fraguela, Flavio F. Marsiglia

**Affiliations:** 1 Department of Psychobiology and Clinical Psychology, Universidade de Santiago de Compostela, 15705 A Coruña, Spain; xa.gomez.fraguela@usc.es; 2 Global Center for Applied Health Research, Arizona State University, Arizona, AZ 85281, USA; Stephanie.L.Ayers@asu.edu (S.L.A.); KULIS@asu.edu (S.S.K.); marsiglia@asu.edu (F.F.M.); 3 Department of Nursing, Universidad de Sevilla, 41004 Sevilla, Spain; imacfadden@us.es

**Keywords:** alcohol use, adolescence, planned behavior, attitudes, resistance strategies

## Abstract

According to the theory of planned behavior (TPB), intentions to perform a specific behavior are the result of attitudes, norms, and perceived control, and in turn, intentions and perceived control are the main predictors of the behavior. This study aimed to test the applicability of TPB in predicting alcohol use in normative pre-adolescents. The sample was composed of 755 Spanish adolescents aged 11 to 15 (*M* = 12.24; SD = 0.56), 47.1% females, from 12 state secondary schools in Spain. The results of path analysis indicate that positive attitudes towards alcohol, favorable norms towards alcohol, and offer vulnerability (perceived control) are significantly positively related to intentions to use alcohol as well as negatively related to actual behavioral control (i.e., actual strategies to avoid alcohol use). In turn, intentions to use and actual control predict higher alcohol frequency and heavy drinking. Significant indirect effects of these antecedents were found on alcohol outcomes through the mediation of intentions and actual control. The findings suggest that the validity and applicability of the TPB in normative pre-adolescents depend on the severity of alcohol use and point to a need to consider negative social influence in decision making processes in early adolescence.

## 1. Introduction

Alcohol consumption among adolescents is a critical area requiring effective prevention and health promotion approaches. Alcohol is the most consumed psychoactive substance among Spanish adolescents (14–18 years), with epidemiological data reporting that 77.9% have consumed alcohol at least once in their lives and more than half of them (58.5%) in the last month [1]. On average, alcohol use in Spain begins at 14 years of age, and weekly use begins at 15.2 years [1]. Decision making is one of the cognitive functions most studied in relation to alcohol consumption in adolescence [2]. Decision making is defined as ability to select a course of action from a set of possible behavioral alternatives [3]. In this regard, if unhealthy habits are acquired in adolescence, the probability that the same behavioral patterns will be maintained in adulthood is increased, and the likelihood of adopting new healthy habits is reduced [4,5]. Considering these alarming facts, prevention of early initiation and substance abuse in adolescence is an important strategy to address increasing rates of alcohol and other drug dependency [6]. The National Strategy on Addictions 2017–2024 points out that prevention in this field should be carried out mainly within the education system and emphasizes the need for evidence-based prevention programs aimed at adolescents [7].

### 1.1. Theory of Planned Behavior

**Figure 1:**
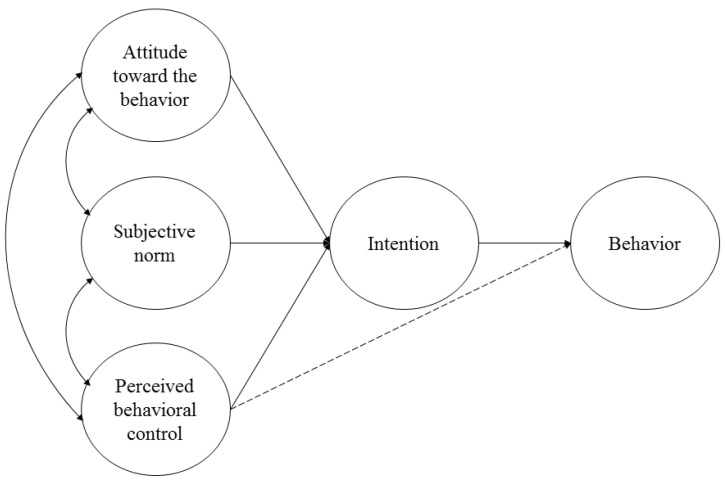
Theory of Planned Behavior [10].

The theory of planned behavior (TPB) [8,9,10] (see Figure 1) has been the dominant theoretical framework to investigate health-related behavior in the past three decades [11]. The TPB is an extension of the theory of reasoned action [12,13]. According to both theories, the most important predictor of a specific behavior is the individual’s intention to perform the behavior, which encompasses the motivational factors that influence a behavior. The TPB [10] proposes that three antecedents are related to intentions: attitudes (favorable or unfavorable attitude toward the behavior; named behavioral beliefs), subjective norms (perceived social pressure; named normative beliefs), and perceived behavioral control (beliefs about the resources and opportunities available that may facilitate or impede the behavior; named control beliefs). The TPB differs from the theory of reasoned action in its addition of perceived behavioral control as an antecedent of intentions to perform the behavior and as a direct predictor of such behavior. Thus, the TPB posits that the influence of attitudes and subjective norms is completely mediated by intentions, while perceived behavioral control, along with intentions, can influence the behavior directly. Moreover, according to both theories, to accurately predict a specific behavior, antecedents and intentions should be specifically related to that particular behavior. The more favorable the attitude and subjective norm and the lower the perceived control, the stronger the intentions are to engage in a behavior.

In the same way that beliefs, motivation, and ability can differently influence a specific behavior [10], they could also differ depending on the specific developmental stage. Previous research has tested the applicability of the TPB to predict alcohol use in pre-adolescents [14], adolescents [15], and mostly in young adults [16]. Although these studies found significant relationships between the components of the TPB and drinking behavior, a recent meta-analytic review concluded that positive attitudes towards alcohol as well as self-efficacy (perceived behavioral control) have a larger association with intentions to use alcohol in adults compared to adolescents [17]. Furthermore, although the TPB has been the reference approach during the last decades, it is not exempt of criticisms that argue for the use of more comprehensive theories [11]. Recent research has proposed that other cognitive, affective, and behavioral components should be taken into account to better explain adolescent health risk behavior [18,19]. These findings suggest the need to review theories and empirically test their applicability in different development stages for improving the efficacy of preventive interventions on problematic behaviors in adolescents. Therefore, this study aims to test the applicability of the TPB on Spanish early adolescents’ alcohol use.

### 1.2. Social Pressure and Negative Peer Influence on Adolescent Alcohol Use

It is well established that the state of health and well-being of adolescents is related to the different environments and relational and ecological contexts to which adolescents belong (families, neighbors, friends, peers, school climate, social, and environmental aspects, etc.) [20]. According to the TPB, the action of the individual is guided, in part, by subjective norms, which are based on normative beliefs about the social pressure to perform the behavior [10]. Social pressure to use substances has been consistently linked to higher levels of substance use in adolescence [21,22] and such pressure comes mainly from peers, who have been posited as a strong influence on drinking behavior from early through late adolescence [23]. Evidence has found that peer’s substance use is specifically associated with adolescent substance use rather than with other types of problematic behavior [21,24,25]. In this regard, offers of alcohol and other substances from intimate friends, relatives, or romantic partners can be particularly influential. This is because those offering are familiar with persuasion triggers, have the opportunity for repeated offers, and can exploit the expectation for maintaining a long-term, reciprocal relationship with the adolescent [26,27,28]. These influential processes make the effective management of refusal strategies more challenging in adolescence [29,30]. Therefore, the offers received can potentially influence the decision-making process of the adolescent to use or not to use alcohol. Taking the offers into account within the TPB model ensures that both active (explicit drug offers) and passive (overestimation of friends’ use; i.e., favorable subjective norms) social pressure is addressed [22]. This more holistic approach is closer to dual-processing approaches that consider a heuristic social-influenced path to risky behavior in addition to the rational reasoning posited in the TPB [19].

### 1.3. Resistance Strategies as a Measure of an Actual Behavioral Control

The incorporation of resistance strategies into the behavioral repertoire of adolescents is implicated in how they resist using substances and avoid risky behaviors successfully [27,31]. Previous research has pointed to the following strategies as the most common behavioral resources used by adolescents [32,33]: “Refuse” offers by saying no verbally or non-verbally; “Explain” why the adolescent does not want to use substances; “Avoid” situations where substances are used; and “Leave” the situations where substances are used. These four strategies are taught in “Mantente REAL” (originally “keepin’ it REAL” [34]), a school-based universal prevention program for substance use and other risk behaviors designed for Secondary Education (middle schools). In line with the TPB [10], the REAL strategies are the resources available to an adolescent that determine, to some extent, the likelihood of alcohol use; that is, actual behavioral control. Thus, the current study further extends the TBP by including actual behavioral control as part of the model to be tested to predict alcohol use in early adolescence. Therefore, this study aims to test the TPB while applying it to the Drug Resistance Strategies (DRS) prevention model used in Mantente REAL. The REAL strategies can be considered as a reflection of actual behavioral control and the offers received by adolescents as a reflection of social pressure to use alcohol.

### 1.4. The Current Study

**Figure 2:**
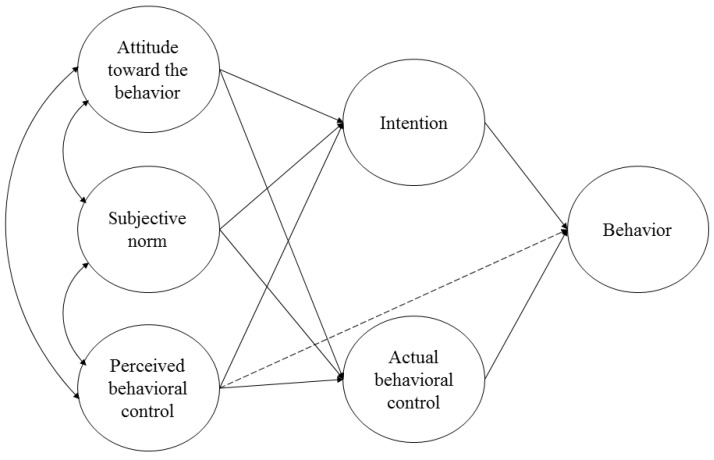
Theory of Planned Behavior Applied to a Drug Resistance Strategies Prevention Model (based on [10]).

This study aims to test the applicability of the TPB in predicting alcohol use in a general pre-adolescent Spanish population and goes beyond the previous research by considering the potential application of the TPB to a DRS prevention model (see Figure 2); that is, considering the role of resistance strategies as a measure of an actual behavioral control. According to the TPB, to accurately predict a behavior, antecedents should be specifically related to such behavior. Therefore, our measures testing TPB are specifically related to alcohol use, except the measure of peer offers that comprises alcohol as well as other drug offers. Furthermore, empirical evidence has demonstrated that negative social influence is a robust risk factor for alcohol use at this specific developmental stage. Hence, in line with a dual-processing approach [19], the current study examines an additional model including offers received as a measure of active social pressure to use alcohol [22]. Considering resistance strategies together with negative social influence in the model can further understanding of the underlying mechanisms of decision making to use alcohol in early adolescence. The overall hypothesis guiding the study from a TPB perspective is that having higher levels of antecedent variables will lead to higher intentions to use alcohol as well as lower behavioral control, both of which leading to higher alcohol use. The current findings might also contribute to the development of prevention guidelines and augment the effectiveness of interventions aimed at preventing adolescent alcohol use in Spain.

## 2. Materials and Methods

### 2.1. Participants and Procedure

The final sample of this study was composed of 755 Spanish adolescents aged 11 to 15 (*M* = 12.24; SD = 0.56), 47.1% females, from Santiago de Compostela (Galicia, NW Spain; 46% of the sample) and Sevilla (Andalucia, S Spain; 54% of the sample). In all, 40.1% of adolescents were born in Galicia, 50.3% in Andalucia, 3.4% in another part of Spain and 6.1% in another country. Almost half the students lived with up to 3 other persons at home (45.5%) and the other half of students reported to live with 4 or more persons (54.5%). Most students reported their parents have a level of education equal to or higher than high school (61.2%) and always have enough money at home for food, transportation, utilities, school fees, and clothes (60.9%). Adolescents were enrolled in the 1st grade of compulsory secondary education in the 2018/2019 school year in 12 state secondary schools, 6 in the region of Santiago and 6 in the city of Sevilla. The schools participated in a test of the “Mantente REAL” drug prevention program [35] (originally “keepin’ it REAL” [34]). Schools were selected by convenience sampling, but randomly assigned to the experimental and control conditions. A total of 934 students were enrolled in the participating schools but 179 of them did not have parental consent, did not assent or were not in school at the first survey. Full information about school recruitment and randomization are reported elsewhere in a test of the intervention’s effects [35]). Because this study is not aimed at analyzing intervention effects, rather controlling for their potential influence on alcohol use, details about the rationale for the intervention are not specified here.

Data were collected from student respondents in the Winter of 2018/2019 (T1) and the Spring 2019 (T2), approximately four months after the pre-test. Self-reported questionnaires were answered by adolescents before and after the program implementation to evaluate variables of interest (the rate of sample attrition between pre- and post-tests was 10.1%). Most of students who answered to both surveys completed more than 80% of the items (97.2% of students in T1 and 85.7% in T2). For the data collection, qualified research team members visited the schools, explained the objectives of the research, and provided proper instructions to the adolescents. Parental consent was requested and, subsequently, adolescent assent was obtained before questionnaires were administered. Adolescent participation was voluntary, and confidentiality of information was completely guaranteed. Compliance with ethical standards was assured throughout the investigation and the project was approved by the Bioethics Committee of the University of Santiago de Compostela and the University Pablo de Olavide.

### 2.2. Instruments

Positive attitudes towards alcohol use (T1): The degree to which adolescents have a favorable or unfavorable evaluation of alcohol use was measured with the item “Is it OK for someone your age to consume alcohol?” Responses were scored from 0 (*definitely not*) to 3 (*definitely yes*).

Favorable subjective norms towards alcohol (T1): The degree to which students perceived social pressure to use alcohol was assessed by asking “Now think about the friends you hang out with. How many do you think have consumed alcohol at least once?” Adolescents responded a scale ranged from 0 (*none of them*) to 5 (*all of them*).

Alcohol offer vulnerability (Perceived behavioral control) (T1): The concept of perceived ease or difficulty to use alcohol was measured as vulnerability to alcohol offers. Adolescents answered to “What would you say if a family member offered you alcohol?” in a response scale scored from 0 (*surely I could say no*) to 3 (*I couldn’t say no*).

Intentions to use alcohol (T1): Adolescents indicated their motivation to use alcohol responding to the item “If you had the chance this weekend, would you consume alcohol?” in a scale ranging from 0 (*definitely not*) to 3 (*definitely yes*).

Actual behavioral control (T1): Adolescents reported the frequency with which they would use specific REAL behavioral strategies to resist alcohol offers (Refuse, saying no directly; Explain, giving reasons to decline; Avoid, not going to places or situations with potential offers; Leave, exiting situations where offers are made; [33]) in the following situation: “If a friend offers you a beer at a party, would you…”. Responses were scored from 0 (*never*) to 4 (*almost always*). The mean score of the four items was used (α = 0.63).

Offers received (T1): The frequency of two types of substance offers were measured: alcohol offers, regardless of who was the offeror (“In the last 30 days, how many times did someone offer you an alcoholic drink?”), and offers received by peers, regardless of the type of substance (“In your life, how many times did a friend or other youth offer you a drug?”). Responses ranged from 0 (*never*) to 4 (*10 times or more*).

Recent alcohol use (T2): Two different measures of alcohol use in the past 30 days were assessed [22,36]. Adolescents reported their frequency of alcohol use (“In the last 30 days, how many times have you had an alcoholic drink?”) and the frequency of heavy drinking episodes (“In the last 30 days, how many times did you drink five or more alcoholic drinks in a row (on the same occasion)?”). Responses were scored from 0 to 6 (*none—only once—2–3 times—4–9 times—10–19 times—20–39 times—40 times or more*).

Covariates: The following variables were assessed at T1 to be included as covariates: “outcome at pre-test” (alcohol use frequency and heavy drinking frequency in T1), “intervention site” (Santiago de Compostela—1 versus Sevilla—0), “intervention condition” (Experimental group—1 versus Control group—0) and “gender” (Male—1 versus Female—0).

### 2.3. Statistical Analyses

**Figure 3:**
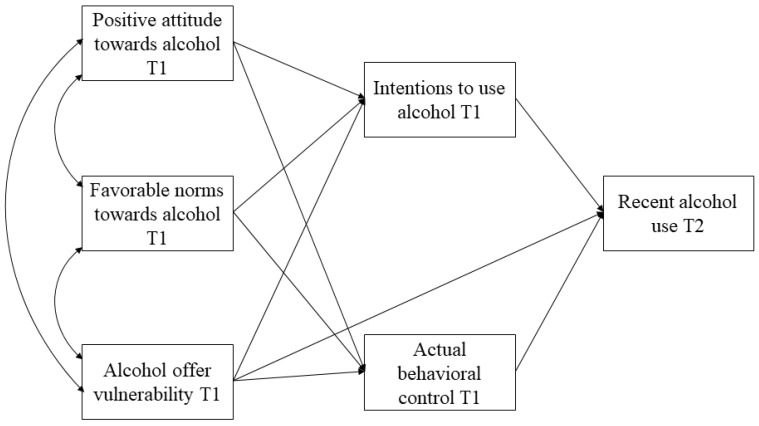
General Path Model Predicting Recent Alcohol Use (Alcohol Frequency and Heavy Drinking Separately).

**Figure 4:**
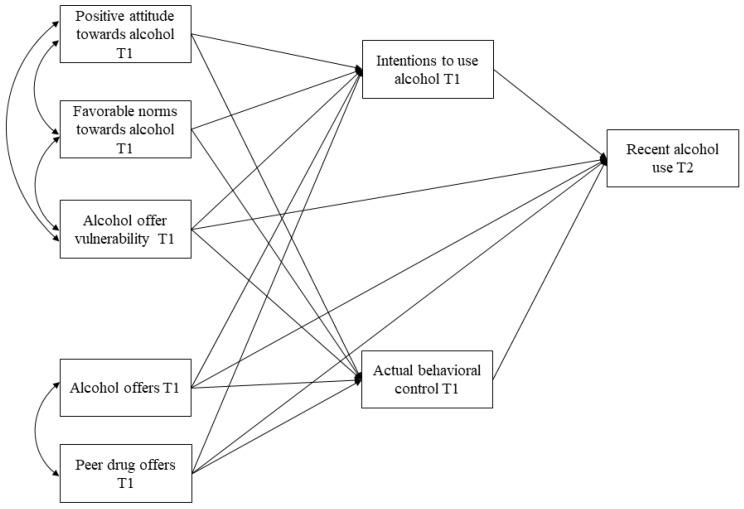
Path Model Predicting Recent Alcohol Use (Alcohol Frequency and Heavy Drinking Separately) Controlling for Negative Social Influence.

IBM SPSS Statistics 24 (IBM, Armonk, NY, USA) and MPLUS 7 (Muthén & Muthén, Los Angeles, CA, USA) were used to conduct the statistical analyses. First, descriptive analyses (means, standard deviations, and range) and bivariate correlations among the study variables were conducted. Next, path analysis models were estimated to examine the relationships between antecedents (i.e., attitudes, norms, and offer vulnerability/perceived control), intentions, actual control, and recent alcohol use (see Figure 3). Mediation effects of antecedents on alcohol use through intentions as well as actual behavioral control were analyzed. Independent models were conducted for each measure of alcohol use (i.e., alcohol frequency and heavy drinking). Path models controlled for outcome at pre-test, city/intervention site, intervention condition, and gender. In addition, to test the potential influence of social pressure in decisions to use alcohol, another two models were estimated controlling also for alcohol offers (regardless of offeror) and peer offers (regardless of substance); these two variables were set to correlate (see Figure 4). All the models were adjusted for the school-level clustering of data and employed full-information maximum likelihood (FIML) estimation to account for attrition to the post-test and item missing data. The robust maximum likelihood method (MLR), which is robust to a non-normal distribution, was used to compute the models and the goodness-of-fit indexes χ^2^/*DF*, CFI, RMSEA, and SRMR were used to evaluate the model fit. The following criteria were considered for an optimum fit: χ^2^/DF < 2–3, CFI > 0.95, RMSEA and SRMR < 0.05; and for an acceptable or reasonable fit: χ^2^/DF < 4, CFI > 0.90, and RMSEA and SRMR < 0.08 [37].

## 3. Results

Table 1 shows descriptive results (means, standard deviations, and ranges) of the main continuous variables of the study for the total sample and both site subsamples. Students reported low means on intentions to use alcohol and its antecedents (positive attitudes towards alcohol, favorable subjective norms towards alcohol, and vulnerability to alcohol offers), as well as low frequencies of alcohol use and, especially, of heavy drinking at both t1 and t2. Students reported having used alcohol for the first time at a mean age of 10.36 (SD = 2.16). Higher means were reported for actual behavioral control; that is, the frequency with which students would use behavioral strategies (Refuse, Explain, Avoid, and Leave) to refuse alcohol offers. Over two-thirds of the total sample (67.8%) reported they would use REAL strategies “often” or “almost always”. Regarding offers, 14% of total students reported having received one or more alcohol offers in the last 30 days and 30.9% received offers of alcohol, tobacco, or other drugs from peers during their lifetime.

Results of bivariate correlations for the total sample (see Table 2) indicated significant and positive intercorrelations among antecedents, intentions, alcohol use, and offers, as well as significant and inverse intercorrelations among all of them with actual behavioral control (use of strategies). After applying the Bonferroni correction, the coefficient between heavy drinking at T1 and actual behavioral control was not significant.

Path analysis was conducted to test the TPB applied to a DRS model (see Figure 3). Several models were analyzed to examine the following relationships: positive attitudes and favorable subjective norms towards alcohol and offer alcohol vulnerability (perceived behavioral control) are directly related to intentions to use alcohol as well as actual behavioral control. Offer vulnerability, intentions and actual control are directly related to alcohol use. Therefore, attitudes, norms, and offer vulnerability can be indirectly related to alcohol use through the mediation of intentions and actual control. Models were tested separately for alcohol frequency and heavy drinking, and after testing a TPB general model, a second model was estimated including controls for negative social influences, as measures by exposure to offers of alcohol and other substances.

The general path model (controlled for intervention site, intervention condition, and gender) did not show adequate overall fit indexes for alcohol frequency (χ^2^ (5) = 71.84, *p* = 0.000; CFI = 0.86; RMSEA = 0.13; SRMR = 0.04), whereas for heavy drinking, CFI and SRMR presented adequate values (χ^2^ (5) = 34.91, *p* = 0.000; CFI = 0.91; RMSEA = 0.09; SRMR = 0.04). Table 3 displays the standardized results of path models. The first three columns of coefficients present the general model for the alcohol frequency outcome. The next column shows the predictors for the heavy drinking outcome, where results predicting intentions and actual control were the same as for alcohol frequency. The remaining columns are for an adjusted model adding negative social influence measures (alcohol offers and peer drug offers), where results predicting intentions and actual control were the same for alcohol frequency and heavy drinking outcomes. Results for the general model indicated, as regards covariates, that gender was significantly related to actual behavioral control and alcohol frequency was predicted by outcome at pre-test, intervention site and intervention condition. Positive attitudes, favorable norms, and offer vulnerability (perceived behavioral control) were significantly and positively related to intentions to use alcohol, as well as negatively related to actual behavioral control (use of REAL strategies). In turn, intentions to use alcohol, positively, and actual behavioral control, negatively, significantly predicted both alcohol frequency and heavy drinking. Offer vulnerability significantly and positively predicted alcohol frequency but not heavy drinking.

Significant indirect effects of antecedents on alcohol use were found for the general model. The results indicated significant mediation effects on alcohol frequency for: attitudes through intentions (β = 0.07, *p* = 0.008) and actual control (β = 0.01, *p* = 0.032); norms through intentions (β = 0.06, *p* < 0.001); and offer vulnerability through intentions (β = 0.11, *p* = 0.015) and actual control (β = 0.02, *p* = 0.032). Significant mediation effects were also found on heavy drinking for attitudes through intentions (β = 0.07, *p* = 0.008) and actual control (β = 0.01, *p* = 0.032); norms through intentions (β = 0.06, *p* < 0.001); and offer vulnerability through intentions (β = 0.11, *p* = 0.015) and actual control (β = 0.02, *p* = 0.032).

On the other hand, the social pressure path model (additionally controlled for alcohol offers and peer drug offers; see Figure 4) fits better than the general model, showing adequate CFI and SRMR indexes for alcohol frequency (χ^2^ (5) = 64.69, *p* = 0.000; CFI = 0.93; RMSEA = 0.13; SRMR = 0.02) and overall adequate fit indexes for heavy drinking (χ^2^ (5) = 30.07, *p* = 0.000; CFI = 0.96; RMSEA = 0.08; SRMR = 0.02). After controlling for negative social influence, the path model showed similar results for covariates, but different significant relationships among the main variables of the model (see Table 3). The results indicated that positive attitudes were only significantly and positively related to intentions to use alcohol and offer vulnerability was significantly related to intentions (positively), actual behavioral control (negatively), and alcohol frequency (positively). In turn, intentions to use alcohol significantly and positively predicted heavy drinking at T2 and actual behavioral control significantly and negatively predicted alcohol frequency at T2. Having received alcohol offers was significantly and positively related to intentions to use alcohol and alcohol frequency, whereas having received drug offers by peers was significantly related to intentions to use alcohol (positively), actual behavioral control (negatively), and both outcomes (positively). In this model, the direct effects of intentions on alcohol frequency and heavy drinking were reduced, while the inclusion of offers received contributed to increase the variance explained by the model.

The results showed fewer significant indirect effects in the social pressure model. Mediation effects on alcohol frequency were found only for offer vulnerability through actual behavioral control (β = 0.01, *p* = 0.005). Mediation effects on heavy drinking were found for attitudes (β = 0.04, *p* = 0.005) and offer vulnerability (β = 0.07, *p* = 0.017) through intentions to use alcohol.

## 4. Discussion

This study aimed to test the applicability of the TPB [10] in predicting alcohol use in a normative pre-adolescent Spanish population within the framework of a DRS prevention model. Thus, the use of REAL resistance strategies has been included as a measure of an actual behavioral control. An additional model has considered the negative social influence on adolescent drinking behavior by including the offers received as a measure of active social pressure.

The results of the general model indicated that positive attitudes towards alcohol, favorable subjective norms, and offer vulnerability are significantly and positively related to intentions to use alcohol as well as negatively related to actual behavioral control (use of REAL strategies) in Spanish pre-adolescents. In turn, intentions to use alcohol and actual behavioral control predict alcohol frequency and heavy drinking. In line with results found in other studies [38], these variables account for a higher explained variance for alcohol frequency (38%) than for heavy drinking (20%). Although most studies testing the applicability of TPB on alcohol use have been conducted with samples of young adults [17], studies have also found that positive attitudes towards use, personal norms, and lack of perceived control significantly predict intentions to use alcohol and other drugs in pre-adolescents [14] and adolescents [15], and that intentions predict last-30 days substance use [14]. Regarding actual behavioral control, the novel component included to test the TPB applied to a DRS prevention model, other studies have similarly found that positive attitudes towards alcohol and favorable subjective norms (positively) as well as perceived behavioral control (negatively) are significantly related to the willingness to use strategies to resist alcohol offers [15]. In turn, using resistance strategies has been found in previous research as a significant factor to reduce the likelihood of engaging in alcohol use [39,40]. To our knowledge no other studies have tested the applicability of the TPB on drinking behavior in Spanish adolescents. Nevertheless, previous research has been conducted on other health-related behaviors in our context, such as healthy sexual behavior (condom use) [41]. That study found, in line with our findings, that positive attitudes towards the behavior, favorable personal norms, and greater perceived control to perform the behavior are significantly related to higher intentions to use condoms. Similarly, intentions to perform the behavior significantly and positively predict the frequency in which young people use condoms during sexual intercourse.

After including active social pressure in the model, the results showed a different pattern of significant relationships. The findings of the current study show that positive attitudes towards alcohol and offer vulnerability seem to be the most robust individual factors related to the mediating variable of intentions to use alcohol since they are significant regardless the presence or not of active social pressure (offers received). These findings are in line with the results of meta-analytic studies that found (experiential) attitudes and self-efficacy as the most robust predictors of intentions to use alcohol [17] and in general health-related behaviors [18]. As a mediator, actual behavioral control was only significantly associated with offer vulnerability when active social pressure is included in the model. This finding indicates the relevant role of the perception of behavioral control or self-confidence on putting in practice the actual behavioral resources to avoid engaging in alcohol use, as stated in the TPB [10] and found in other studies [42].

In this integrated model that considers social pressure, alcohol frequency, and heavy drinking show different predictors: offer vulnerability and actual behavioral control (resistance strategies) predict alcohol frequency, while intention to use predicts heavy drinking. Other studies have found non-significant relationships between perceived behavioral control and heavy patterns of alcohol use in undergraduate youth [43]. Moreover, significant mediation effects on alcohol frequency appear only for offer vulnerability through actual behavioral control, whereas attitudes and offer vulnerability become significant predictors of heavy drinking through the mediation of intentions to use alcohol. Previous research has identified the presence of differential significant predictors based on the pattern of consumption. Cooke et al. [17] found in their meta-analytic study that perceived behavioral control and self-efficacy have significantly stronger relationships with intentions to use alcohol in youth who show light episodic drinking, compared to those who present heavy episodic drinking. In line with the current results, it seems that the variables related to behavioral control (perceived or actual) strongly influence light patterns of alcohol use (alcohol frequency) and intentions seem to strongly influence heavy patterns of alcohol use.

The current results indicate that the general TPB model does not fit very well for frequency of alcohol use, but it does fit better for heavy drinking. These findings suggest that the TPB does not reflect the processes in pre-adolescence for forms of antisocial behavior that are “more normative” or less severe (alcohol frequency) because they are influenced more by social pressure than by individual or cognitive processes (main components of the TPB). In this regard, previous studies have found that progression to problematic alcohol use patterns in young adulthood are robustly predicted by conduct problems, sensation seeking, and illicit substance use, together with antisocial behavior in the peer group [44]. Similarly, Guillén, Roth, Alfaro, and Fernández [45] found that adolescents with problematic alcohol use present significantly higher levels of antisocial behavior and that those adolescents with lower levels of alcohol use report more frequent exposure to high peer pressure. Other studies have found that impulsivity and sensation seeking in tenth-grade adolescents are more strongly predictive of binge drinking one year later than of alcohol use overall [46]. These findings suggest that peer pressure is sufficient to influence experimentation with alcohol use, whereas an individual/antisocial behavioral pattern may be also needed to show heavy and problematic drinking behavior.

It is relevant to note that considering social pressure in the model (i.e., alcohol offers regardless of the relationship to the offeror and peer offers regardless of the substance) increases the fit of the model as well as the variance explained, especially for alcohol frequency, which can be considered a less severe pattern of use than heavy drinking. Along this line, other studies have highlighted the need to consider social (parent and peer) influences on adolescent drinking behavior because they account for a substantial percentage of variance in the models predicting alcohol use at this developmental stage [15,23]. The notable changes in the current results depending on having considered social pressure in the model are, in part, due to the strong effects that antisocial influences have on intentions and behavior in adolescence, especially if it comes from peers [21,38].

Although the TPB appears to be applicable to a DRS prevention model (such as Mantente REAL) for predicting alcohol use in normative Spanish pre-adolescents, this theory is limited in explaining differences in drinking behavior depending on its severity. This conclusion supports other previous research suggesting the need for more comprehensive approaches to better explain health adolescent behavior [11]. To better understand the underlying mechanisms of decision making in light use or heavy use of alcohol in early adolescence, a more holistic dual-processing model may be needed. A dual-processing approach could be particularly appropriate for adolescent behavior as it considers the role of a heuristic system, more experiential, superficial, and quick, that is more typical for the developmental adolescent stage than the rational, analytic, and in-depth system underlying the TPB [19]. Considering the findings of the current study, social pressure to use alcohol seemed to capture the more reactive, socially-oriented path of the heuristic process [19]. It might be that social pressure might influence the heuristic processing more strongly, leading to the onset of light patterns of alcohol use, while individual characteristics might influence rational processing more strongly and the escalation to more heavy patterns of alcohol use. A dual-processing model might also help to understand whether some mediators may be more central to delaying initiation of alcohol use (prolonging abstinence; in this case, actual behavioral control by using resistance strategies), while other mediators may be crucial to changing the drinking behavior of prior users (in this case, intentions to use alcohol more or less heavily).

Taken the current findings together, some prevention guidelines can be drawn for augmenting the effectiveness of interventions aimed at preventing alcohol use in Spanish adolescents. As heavy drinking patterns are less common at pre-adolescent ages in Spanish youth [1,35], prevention programs should focus on preventing the initial patterns of light alcohol use by strengthening behavioral control and self-efficacy perceptions in adolescents. As the current findings suggest, teaching pre-adolescents resistance strategies could prevent or delay the initial onset of alcohol use, and, consequently, prevent the escalation to more problematic patterns of alcohol use as youth transition into young adulthood [47,48]. Prevention interventions can augment their effectiveness when the programs focus on changing the empirically-robust mediators (e.g., resistance skills, general assertiveness, personal competence, normative perceptions of substance use) and not only the intervention’s main outcomes [49], as it occurs in Mantente REAL [34]. Because alcohol use is much more prevalent in middle and late adolescence than in early adolescence [50,51], implementing prevention programs such as Mantente REAL with pre-adolescents might work principally by facilitating the acquisition of behavioral strategies to effectively cope with peer pressure in later ages. In this regard, evidence has supported resistance strategies and refusal self-efficacy as effective skills to avoid engaging in substance use during adolescence [49].

The current study presents some limitations that should be considered for the proper interpretation of the findings. First, findings from path analysis do not prove causal relationships. Furthermore, the antecedents of the behavior in the model were assessed only by one general item each, which does not provide information about more specific constructs influencing behavior (e.g., experiential vs. instrumental attitudes). Another limitation is the representativeness of the sample. Although it is a large sample, it is not designed to represent all of Spanish territory in its regional diversity and complexity. Moreover, the models employed in the study are not exhaustive and they could be expanded with other variables. For instance, the models could incorporate macro-level influences, such as cultural determinants (e.g., access to substances or perception of risk depending on cultural norms) that influence conditioning factors like social pressure to use alcohol, and attitudes, norms, and perceived control regarding its use. Nor do the models incorporate individual-level variables that may be important influences on intentions, such as temperament or personality. Future studies should consider testing the TPB as well as the dual-processing approach in sample subgroups (e.g., gender, age of drinking onset, and impulsivity orientation) that may inform different patterns of response to the effectiveness of prevention programs. Finally, a longitudinal study with more medium- and long-term follow-up data would provide clearer and more solid evidence of the mediating processes posited by the TPB and how they unfold across the transition from pre-adolescence to young adulthood.

## 5. Conclusions

The findings of this study make an important contribution to the existing literature about youth’s alcohol use in Spain and its prevention as guided by the Theory of Planned Behavior perspective. The current results advance prevention science from a developmental perspective by differentiating more clearly between the alcohol prevention needs of preadolescents and adolescents. One of the main findings of this study is that preadolescent alcohol prevention interventions should focus on social pressure more than individual or cognitive processes. The social and normative nature of alcohol initiation and consumption among preadolescents in Spain highlights the need for training in the resistance strategies that would allow youth to remain in the group while avoiding the use of alcohol.

## Figures and Tables

**Table 1:** Descriptive Statistics of the Main Variables of the Study for the Total Sample and for the Both Cities.

Variables	Total Sample	Santiago Sample	Sevilla Sample	
	*M (SD)*	*M (SD)*	*M (SD)*	Range
Positive attitudes	0.14 (0.38)	0.12 (0.32)	0.15 (0.42)	3
Favorable norms	0.56 (1.04)	0.40 (0.89)	0.70 (1.13)	5
Offer vulnerability	0.27 (0.61)	0.23 (0.58)	0.30 (0.63)	3
Intentions	0.14 (0.49)	0.10 (0.39)	0.18 (0.56)	3
Actual control	1.55 (0.84)	1.62 (0.82)	1.49 (0.87)	3
Alcohol frequency T1	0.15 (0.59)	0.12 (0.49)	0.24 (0.66)	6
Heavy drinking T1	0.04 (0.26)	0.02 (0.16)	0.05 (0.32)	6
Alcohol frequency T2	0.30 (0.76)	0.17 (0.63)	0.42 (0.85)	6
Heavy drinking T2	0.06 (0.29)	0.03 (0.23)	0.08 (0.33)	6
Alcohol offers	0.24 (0.70)	0.15 (0.55)	0.31 (0.80)	4
Peer drug offers	0.31 (0.64)	0.21 (0.49)	0.39 (0.74)	4

**Table 2:** Bivariate Correlations Between the Main Variables of the Study for the Total Sample.

Variables	Positive Attitudes	Favorable Norms	Offer Vulnerability	Intentions	Actual Control	Alcohol Freq T1	Heavy Drink T1	Alcohol Freq T2	Heavy Drink T2	Alcohol Offers	Peer Drug Offers
Positive attitudes	1										
Favorable norms	0.40 ***	1									
Offer vulnerability	0.37 ***	0.48 ***	1								
Intentions	0.46 ***	0.49 ***	0.57 ***	1							
Actual control	−0.22 ***	−0.27 ***	−0.33 ***	−0.27 ***	1						
Alcohol freq. t1	0.35 ***	0.46 ***	0.38 ***	0.59 ***	−0.20 ***	1					
Heavy drinking t1	0.21 ***	0.31 ***	0.27 ***	0.44 ***	−0.11 **^,a^	0.50 ***	1				
Alcohol freq. t2	0.31 ***	0.38 ***	0.39 ***	0.45 ***	−0.21 ***	0.53 ***	0.17 ***	1			
Heavy drinking t2	0.21 ***	0.29 ***	0.30 ***	0.37 ***	−0.17 ***	0.41 ***	0.15 ***	0.60 ***	1		
Alcohol offers	0.30 ***	0.47 ***	0.34 ***	0.44 ***	−0.19 ***	0.49 ***	0.37 ***	0.42 ***	0.32 ***	1	
Peer drug offers	0.39 ***	0.52 ***	0.39 ***	0.49 ***	−0.26 ***	0.50 ***	0.32 ***	0.46 ***	0.33 ***	0.53 ***	1

^a^ Non-significant after applying the Bonferroni correction to account for the multiple comparisons. ** *p* < 0.01. *** *p* < 0.001.

**Table 3:** Standardized Results of Path Analysis Models Predicting Alcohol Frequency and Heavy Drinking at T2.

Variables	General Model				Social Pressure Model			
	Intentions	Actual Control	Alcohol Frequency T2	Heavy Drinking T2	Intentions	Actual Control	Alcohol Frequency T2	Heavy Drinking T2
Intercept	−0.08	1.96 ***	0.45 ***	0.32 ***	−0.12 **	1.98 ***	0.32 ***	0.17
Outcome pretest			0.41 ***	0.08			0.31 ***	0.01
Intervention site	−0.02	0.04	−0.11 ***	-0.06	0.00	0.03	−0.09 ***	−0.04
Intervention condition	0.00	−0.01	−0.05 **	−0.06	−0.00	−0.01	−0.06 ***	−0.06
Gender	0.01	0.05 **	−0.01	−0.01	0.01	0.05 **	0.00	−0.00
Positive attitudes	0.24 ***	−0.08 *			0.19 ***	−0.06		
Favorable norms	0.21 ***	−0.11 *			0.09	−0.07		
Offer vulnerability	0.38 ***	−0.24 ***	0.17 **	0.13	0.34 ***	−0.23 ***	0.14 *	0.09
Intentions			0.15 *	0.30 **			0.09	0.21 **
Actual control			−0.06 ***	−0.07 *			−0.04 ***	−0.05
Alcohol offers					0.14 *	0.02	0.11 *	0.13
Peer drug offers					0.18 *	−0.11 *	0.18 ***	0.17 **
R2	0.42	0.13	0.38	0.20	0.47	0.14	0.44	0.24

* *p* < 0.05; ** *p* < 0.01; *** *p* < 0.001.
